# Cerebrospinal Fluid Biomarkers in Relation to MRZ Reaction Status in Primary Progressive Multiple Sclerosis

**DOI:** 10.3390/cells9122543

**Published:** 2020-11-25

**Authors:** Tilman Robinson, Ahmed Abdelhak, Tanima Bose, Edgar Meinl, Markus Otto, Uwe K. Zettl, Rick Dersch, Hayrettin Tumani, Sebastian Rauer, André Huss

**Affiliations:** 1 Clinic of Neurology and Neurophysiology, Medical Center—University of Freiburg, Faculty of Medicine, University of Freiburg, 79085 Freiburg, Germany; tilman.robinson@uniklinik-freiburg.de (T.R.); rick.dersch@uniklinik-freiburg.de (R.D.); sebastian.rauer@uniklinik-freiburg.de (S.R.); 2 Department of Neurology, University Hospital Ulm, 89081 Ulm, Germany; ahmed.abdelhak@uni-tuebingen.de (A.A.); markus.otto@uni-ulm.de (M.O.); andre.huss@uni-ulm.de (A.H.); 3 Biomedical Center and Klinikum Grosshadern, Institute of Clinical Neuroimmunology, Ludwig Maximilian University, 81377 Munich, Germany; Tanima.Bose@med.uni-muenchen.de (T.B.); edgar.meinl@med.uni-muenchen.de (E.M.); 4 Neuroimmunological Section, Department of Neurology, Medical Center of the University of Rostock, 18051 Rostock, Germany; uwe.zettl@med.uni-rostock.de; 5 Specialty Hospital Dietenbronn, 88477 Schwendi, Germany

**Keywords:** MRZ reaction (MRZR), primary progressive multiple sclerosis (PPMS), B cell-activating factor (BAFF), chemokine CXC ligand 13 (CXCL-13), soluble B cell maturation antigen (sBCMA), soluble transmembrane activator and CAML interactor (sTACI), chitinase-3-like protein 1 (CHI3L1), glial fibrillary acidic protein (GFAP), neurofilament light chain (NfL)

## Abstract

The MRZ reaction (MRZR) comprises the three antibody indices (AIs) against measles, rubella, and varicella zoster virus, reflecting an intrathecal polyspecific B cell response highly specific for multiple sclerosis (MS). Thus, MRZR can be used to confirm a diagnosis of primary progressive MS (PPMS) but its pathophysiological and wider clinical relevance is unclear. This study aimed to investigate whether PPMS patients with a positive MRZR (MRZR+) differ from those with a negative MRZR (MRZR-) according to cerebrospinal fluid (CSF) biomarkers of B cell activity, neuroaxonal damage or glial activity, and clinical features. (1) Methods: In a multicenter PPMS cohort (*n* = 81) with known MRZR status, we measured B cell-activating factor (BAFF), chemokine CXC ligand 13 (CXCL-13), soluble B cell maturation antigen (sBCMA), soluble transmembrane activator and CAML interactor (sTACI), and chitinase-3-like protein 1 (CHI3L1) in the CSF with enzyme-linked immunosorbent assays (ELISAs). Glial fibrillary acidic protein (GFAP) and neurofilament light chain (NfL) were detected in serum and CSF using single molecule array (SIMOA) technology. (2) Results: MRZR+ patients (45.7% of all PPMS patients) revealed higher levels of NfL in CSF compared to MRZR- patients (54.3%). There were positive correlations between each of sBCMA, sTACI, and intrathecal immunoglobin G (IgG) synthesis. Additionally, NfL concentrations in serum positively correlated with those in CSF and those of GFAP in serum. However, MRZR+ and MRZR- patients did not differ concerning clinical features (e.g., age, disease duration, Expanded Disability Status Scale (EDSS) at diagnosis and follow-up); CSF routine parameters; CSF concentrations of BAFF, CXCL-13, sBCMA, sTACI, CHI3L1, and GFAP; or serum concentrations of GFAP and NfL. (3) Conclusions: In PPMS patients, MRZR positivity might indicate a more pronounced axonal damage. Higher levels of the soluble B cell receptors BCMA and transmembrane activator and CAML interactor (TACI) in CSF are associated with a stronger intrathecal IgG synthesis in PPMS.

## 1. Introduction

It has been found that 10 to 15% of all multiple sclerosis (MS) patients are suffering from primary progressive multiple sclerosis (PPMS) [1]. In contrast to the more common primary relapsing–remitting type of MS (RRMS), PPMS patients show a more balanced gender distribution, later symptom onset, and gradually increasing neurological disability without discernable relapses [2,3]. B cells and humoral immune mechanisms have been suggested as playing a key role in the pathogenesis of MS [4,5,6]. This hypothesis is supported by the efficacy of ocrelizumab, a humanized monoclonal antibody that selectively depletes CD20-expressing B cells and the only MS drug currently approved for both RRMS and PPMS patients [7,8]. In PPMS, the observed diffuse demyelination and axonal loss but less overt inflammation might indicate an antibody-mediated pathogenesis [9,10]. Intrathecal immunoglobulin G synthesis was recently found to be associated with disability worsening in MS [11]. Another characteristic feature of the humoral immune response in MS is the intrathecal polyspecific autoantibody production against different antigens in the cerebrospinal fluid (CSF), which seems to be very specific for MS [12]. This phenomenon is known as the MRZ reaction (MRZR; according to the three most frequently found antibody targets in the CSF of MS patients: measles, rubella, and varicella zoster virus) [13,14]. It has been shown that MRZR is at least as prevalent and highly specific for PPMS as it is for RRMS and therefore worthy of consideration to improve diagnosis in cases of suspected PPMS [15,16]. Further information on the clinical and pathophysiological implications of MRZR in PPMS might potentially be helpful. Therefore, this exploratory study was conducted to investigate whether and how PPMS patients, depending on their MRZR status (positive MRZR result (MRZR+) versus negative MRZR result (MRZR-)), differ regarding clinical disease severity and CSF biomarkers of B cell activity, neuroaxonal damage (neurofilament light chain (NfL)) and glial activity (chitinase-3-like protein 1 (CHI3L1) and glial fibrillary acidic protein (GFAP)). As the pattern of B cell biomarkers might shed more light on the pathophysiological role of MRZ reaction in particular, we measured four different B cell biomarkers: soluble B cell maturation antigen (sBCMA), soluble transmembrane activator and CAML interactor (sTACI), B cell-activating factor (BAFF), and chemokine CXC ligand 13 (CXCL-13).

### 1.1. Patients

All 81 patients enrolled in this study derived from a multicenter, retrospective PPMS cohort (*n* = 93) [15]. The only selection criteria, not met by 12 PPMS patients, were that the MRZR result was known and that a sample of the same CSF and serum used for MRZR determination was available. In all patients, the diagnosis of PPMS had been reviewed, applying the 2017 McDonald criteria, after careful exclusion of relevant differential diagnoses [17]. In each case, lumbar puncture (LP) had been performed as part of the diagnostic routine so that the time of LP coincided with the diagnosis of PPMS. Clinical disease severity was assessed using the Expanded Disability Status Scale (EDSS) [18]. Disease course was characterized by development of EDSS and clinical disease progression, defined as documented increasing neurological dysfunction/disability without unequivocal recovery within the two years prior to LP [19]. These parameters were obtained from medical records.

### 1.2. CSF and Serum Routine Parameters

All paired CSF and serum samples were collected on the same day and stored at minus 80 °C according to the consensus protocol for the standardization of CSF collection and biobanking [20]. Hemolytic CSF samples were excluded. CSF and serum routine parameters were measured in the local laboratory of the corresponding university hospital, as reported in detail previously [15]. CSF leukocyte count was determined using the Fuchs Rosenthal Counting Chamber (Hecht, Sondheim, Germany). Total immunoglobulin concentrations in the serum and the CSF were detected nephelometrically (ProSpect System, Siemens, Erlangen, Germany). Levels of measles, rubella, and varicella immunoglobin G (IgG) (IgGspec) in CSF and serum were measured using enzyme-linked immunosorbent assay (ELISA; Serion classic ELISA, Germany) followed by MRZR determination, as described previously using the threshold of at least two positive antibody indices (AIs ≥ 1.5) for a positive MRZR result (MRZR-2 definition) [16]. In cases where an antibody index (AI) could not be calculated due to non-detectable antibodies in the CSF against measles, rubella, or varicella zoster virus, we considered AI to be 1.0 (negative) [12,16,21]. Quantitative intrathecal Ig synthesis was assessed using the IgG index (Q_IgG_/Q_Alb_), IgG_loc_, and the intrathecal synthesis for IgG/A/M as a percentage according to the Reiber formulas [22]. Negative results of IgG_loc_ were considered to be zero. Intrathecal Ig synthesis as a percentage was considered to be absent for values less than 10% [22]. Furthermore, for a qualitative assessment of intrathecal IgG synthesis, we detected oligoclonal IgG bands (OCB) by performing isoelectric focusing on agarose gel followed by immunofixation (Hydragel Isofocusing, Sebia, Lisses, France). A positive OCB result was defined as two or more OCB in the CSF [23].

### 1.3. BAFF, CXCL-13, GFAP, CHI3L1, NfL, sBCMA, and sTACI

GFAP and NfL in CSF and serum were measured using single molecule array (SIMOA) assays (GFAP Discovery kits and NfL Early Access assays, Quanterix Corporation, Billerica, MA, USA). The other biomarkers were detected in CSF using commercial ELISA by R&D Systems (BAFF: DBLYS0B; CXCL-13: DCX130; CHI3L1: DC3L10; sBCMA: DY193; and sTACI: DY174). Results of measurements of GFAP, CHI3L1, and NfL in the same PPMS cohort have been reported separately [24].

### 1.4. Statistical Analysis

All statistical analyses were performed using Graph Pad Prism 6 (Graph Pad Software Inc., La Jolla, CA, USA). Comparison of MRZR+ and MRZR- PPMS patients on categorical data was performed using Fisher’s exact test. For interval scale data, the unpaired *t*-test was applied, except for data failing the Shapiro–Wilk test, for which the Mann–Whitney *U* test was used. Calculation of correlation matrices for age, EDSS, and disease duration at the time of LP; MRZ-AIs; concentrations of CXCL-13, BAFF, sBCMA, sTACI, GFAP, CHI3L1, and NfL; as well as CSF leucocyte count and IgG_loc_ were performed with Spearman’s rho test with Holm–Bonferroni correction for multiple comparisons. A two-tailed *p*-value ≤ 0.05 was considered as statistically significant. In this paper, all uses of “significant” refer to statistically significant.

## 2. Results

### 2.1. MRZ Reaction

In total, 81 PPMS patients were included in this study, 56 of them were treated at the Medical Center—University of Freiburg, 24 at the Department of Neurology of the university hospital of Ulm, and 1 was treated at the university hospital of Rostock. Of the 81 PPMS patients, 45.7% were MRZR+ (22.2% with three positive AIs and 23.5% with two positive AIs) and 54.3% were MRZR- (32.1% with one positive AI and 22.2% without any positive AI). The presence of MRZR positivity did not differ significantly between medical centers of Freiburg and Ulm (*p* = 0.147). Mean AI values of the entire PPMS group were 3.2 for measles (M), 2.7 for rubella (R), and 2.5 for varicella zoster virus (Z); the frequency of positive AI was 46.9% for M, 42.0% for R, and 46.9% for Z. In 9.5% of all measurements of antibodies against M, R, or Z in CSF, antibodies against a virus were not detectable.

### 2.2. Clinical Features

The entire PPMS group exhibited an almost balanced gender distribution (54.4% females) and a mean age of 43.2 years (range: 15–62 years; SD: 10.2) at the time of first MS symptoms and of 50.8 years (25–78; 10.9) at the time of LP with a mean disease duration of 7.4 years (1–39; 7.7). The clinical disease severity assessed by the EDSS at the time of LP was 4.7 (1.5–8.5; 1.7) and 5.6 (1.5–8.0; 1.7) at last follow-up (mean follow-up duration: 28.8 months ranging from 2 to 80 months; SD: 24.2). The mean individual EDSS change between these two time points was +1.0 (ranging from −0.5 to +4.5; SD: 1.3). The majority of PPMS patients (88.1%) had been clinically progressive within the two years prior to LP. As shown in Table 1, all clinical features did not differ significantly between MRZR+ and MRZR- PPMS patients.

### 2.3. CSF Routine Parameters

None of the CSF routine parameters differed significantly between MRZR+ and MRZR- PPMS patients, as presented in Table 2. Out of six PPMS patients who tested negative for OCB (OCB-), four (66.7%) exhibited a positive MRZR. None of the six OCB- PPMS patients displayed any other inflammatory alterations in CSF such as CSF pleocytosis or any intrathecal Ig synthesis.

### 2.4. B Cell Biomarkers in CSF

**Figure 1:**
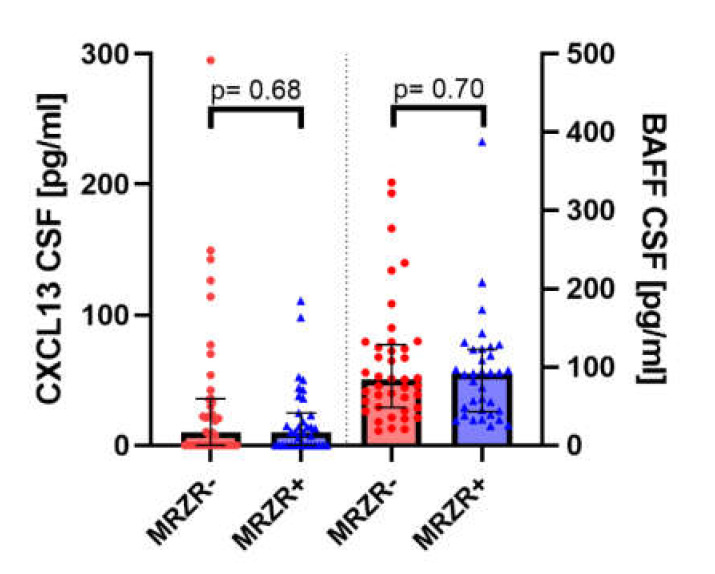
CXCL-13: chemokine CXC ligand 13; CSF: cerebrospinal fluid; BAFF: B cell-activating factor; MRZR-: primary progressive multiple sclerosis patients with no more than one positive MRZ antibody index; MRZR+: primary progressive multiple sclerosis patients with at least two positive MRZ antibody indices.

Detectable concentrations were found in 100% of CSF samples for BAFF and in 42.1% for CXCL-13 (more details in Table 3). MRZR+ and MRZR- PPMS patients had similar CSF concentrations of both BAFF (*p* = 0.70) and CXCL-13 (*p* = 0.68), as shown in Figure 1. This picture did not change when only patients with three positive MRZ-AIs (*n* = 18) were compared with those without any positive MRZ-AIs (*n* = 18; data not shown).

**Figure 2:**
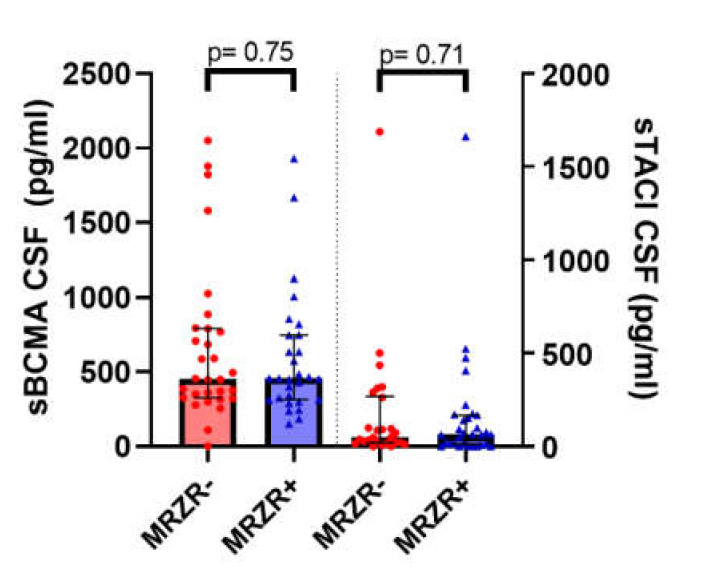
sBCMA: soluble B cell maturation antigen; CSF: cerebrospinal fluid; sTACI: soluble transmembrane activator and CAML interactor; MRZR-: primary progressive multiple sclerosis patients with no more than one positive MRZ antibody index; MRZR+: primary progressive multiple sclerosis patients with at least two positive MRZ antibody indices.

The detection rate in CSF samples was 98.3% for sBCMA and 86.0% for sTACI (more details in Table 3). MRZR+ and MRZR- PPMS patients did not significantly differ regarding CSF concentrations of either sBCMA (*p* = 0.75) or sTACI (*p* = 0.71), as illustrated in Figure 2. These findings did not change when only patients with three positive MRZ-AIs were compared with those without any positive MRZ-AIs (data not shown).

Levels of the four B cell biomarkers (BAFF, CXCL-13, sBCMA, and sTACI) neither differed significantly between clinical progressive (*n* = 59) and non-progressive (*n* = 8) patients nor between patients with (*n* = 11) and without EDSS progression (*n* = 9) until follow-up or between patients who underwent LP after less (*n* = 15) and more than one year disease duration (*n* = 62) (data not shown).

### 2.5. Biomarkers of Glial Activation and Neuroaxonal Damage in CSF and Serum

MRZR+ PPMS patients had higher levels of NfL in CSF (median: 1720 pg/mL) than MRZR- patients (median: 1110 pg/mL; *p* = 0.04), as presented in Table 4. In contrast, concentrations of CHI3L1 in CSF, NfL in serum, as well as GFAP in serum and CSF were similar in MRZR+ and MRZR- patients. Levels of NfL (in serum and CSF), GFAP (in serum and CSF), and CHI3L1 neither differed significantly between clinical progressive and non-progressive patients nor between patients with and without EDSS progression until follow-up or between patients with less and more than one year disease duration at the time of LP (data not shown).

### 2.6. Correlation Analysis

The analysis revealed no significant correlation of any of the three AIs for measles, rubella, and varicella zoster virus with any of the other CSF biomarkers, with CSF leucocyte count, intrathecal IgG synthesis (IgG_loc_), or any clinical parameters (age, EDSS, disease duration). Clearly positive correlations were found between sTACI and intrathecal IgG synthesis (IgG_loc_) (ρ = 0.79; *p* < 0.001), between sBCMA and sTACI (ρ = 0.70; *p* < 0.001), and between sBCMA and IgG_loc_ (ρ = 0.65; *p* = 0.005). Positive correlations were also observed between NfL in serum and in CSF (ρ = 0.64; *p* < 0.001), between NfL and GFAP both in serum (ρ = 0.51; *p* = 0.001), as well as between GFAP in serum and age at the time of LP (ρ = 0.43; *p* = 0.043). There was a weakly positive correlation between the AIs of measles and rubella (ρ = 0.40; *p* = 0.031). All other correlations were not significant after Holm–Bonferroni correction.

As B cell biomarkers were of special interest in this study, correlations including a B cell biomarker with ρ > 0.3 are mentioned here despite not being significant after Holm–Bonferroni correction: CXCL-13 and sBCMA (ρ = 0.41; *p* = 0.21), CXCL-13 and sTACI (ρ = 0.38; *p* = 0.53), CXCL-13 and CSF cell count (ρ = 0.34; *p* = 0.62), and CXCL-13 and IgG_loc_ (ρ = 0.37; *p* = 1.88).

EDSS and disease duration at LP showed no significant correlations with any of the biomarkers; however, the correlation between age and disease duration at LP (ρ = 0.39; *p* = 0.056) as well as between disease duration and EDSS at LP (ρ = 0.4; *p* = 0.061) only just failed to reach significance.

## 3. Discussion

### 3.1. CSF Biomarkers for MS Diagnosis

In this multicentric PPMS cohort, we report a positive MRZR in around half of PPMS patients overall and in each treatment center. Our finding that a considerable proportion (66.7%) among the few OCB- PPMS patients were MRZR+ is in line with earlier reports predominantly concerning RRMS patients [25,26]. Furthermore, in all MRZR+OCB- PPMS patients, positive MRZR was the only available sign of CSF inflammation, highlighting its diagnostic value in those rare cases of suspected MS without OCB. In the last revision of the McDonald criteria, OCB are the only CSF biomarker explicitly mentioned for use in MS diagnosis [17]. The high specificity of MRZR and the high sensitivity of OCB illustrate the complementary role of these two CSF biomarkers of MS, suggesting their combined use to achieve a higher level of diagnostic accuracy [12,26,27,28,29].

### 3.2. CSF Biomarkers as Indicators for Clinical Disease Severity and Disease Course in PPMS

The specificity of MRZR for MS makes this biomarker relevant for confirming a MS diagnosis, and raises questions about possible other implications of an MRZR result. What is known is that in patients with clinical isolated syndrome, suggesting MS, MRZR has a high positive predictive value for the conversion into definitive RRMS [28,30]. However, at least according to the present study, MRZR does not seem to be clearly associated with clinical disease severity or disease course in PPMS. The same apparently applies to a number of other CSF biomarkers (CXCL-13, BAFF, sTACI, sBCMA, NfL, GFAP, and CHI3L1), none of which showed a significant correlation with EDSS. The low proportion of follow-up EDSS data (25%) of the present PPMS cohort may have been a limiting factor, as correlations between EDSS and CSF biomarkers have been found in some studies (mainly of RRMS patients), for example between EDSS and CXCL-13 [31]. However, in a slightly smaller PPMS cohort, there was no correlation between NfL and EDSS, in line with the present results [32]. This is supported by another MS cohort consisting of 75 RRMS and 12 progressive MS patients in which EDSS did not correlate with NfL or GFAP in CSF [33]. For RRMS patients, it has been shown that initially MRZR- patients can occasionally turn MRZR+ over time but apparently not the other way around [34]. Given this finding, the present observation that MRZR+ PPMS patients were not older or suffering from longer disease duration than MRZR- patients might imply that the rate of conversion from MRZR- into MRZR+ within the disease course is possibly not as high in PPMS as in RRMS. Alternatively, conversion from MRZR- to MRZR+ occurs in earlier stages of the disease course of PPMS before LP is done.

### 3.3. CSF Biomarkers in Relation to MRZR

The most cited hypothesis about the physiology of MRZR is that it is a result of a non-specific bystander activation of B cells [12]. Two cytokines involved in survival and attraction of B cells into the CNS in neuroinflammation are B cell-activating factor (BAFF) and chemokine CXC ligand 13 (CXCL-13), which have been studied in the CSF of MS patients [31,35,36,37,38,39]. The regulation of B cell homeostasis involves two ligands (BAFF and a proliferation-inducing ligand (APRIL) [40]) and three receptors, of which B cell maturation antigen (BCMA) and transmembrane activator and CAML interactor (TACI) are both detectable in soluble form in CSF, reflecting compartmentalized B cell accumulation and activation [41,42]. These B cell biomarkers have not yet been studied in relation to MRZR in MS patients; neither have NfL, GFAP, or CHI3L1 [43,44].

The present study results did not support the hypothesis that MRZR positivity might be an indicator of a higher B cell activity, as MRZR+ and MRZR- PPMS patients neither differed in the level of any of the four B cell biomarkers studied (sBCMA, sTACI, CXCL-13, and BAFF), nor in respect of other signs of generally more pronounced B cell activity (such as higher CSF cell count or higher intrathecal Ig synthesis). This picture was additionally confirmed by the absence of positive correlations between the three MRZ AIs and any of the studied B cell biomarkers. Nevertheless, clearly positive correlations between each of the two B cell receptors studied (sBCMA, sTACI) and intrathecal IgG synthesis were found, consistent with the view that these B cell receptors are derived from antibody-secreting plasma cells and plasmablasts in the CSF [45]. To our knowledge, until now there has been no data regarding sBCMA or sTACI in the CSF of PPMS patients. However, sBCMA and sTACI have recently been shown to be elevated in the CSF of RRMS patients compared to controls and to correlate with the IgG index and interestingly also with concentration of sCD27 [41,46]. One of the very few previous studies addressing differences between MRZR+ and MRZR- MS patients reported higher concentrations of Ig kappa-free light chains in MRZR+ RRMS patients [47].

A main contribution of the present PPMS study is to have shown that MRZR+ patients exhibit higher levels of NfL in CSF compared to MRZR- patients. NfL is exclusively expressed in neurons and forms part of the neuronal cytoskeleton [48]. It can be released into extracellular space when neuroaxonal damage occurs and has therefore been proposed as a biomarker for neurodegenerative processes in MS [49,50]. CSF concentrations of NfL have been reported to be generally increased in MS compared to healthy controls and even higher in non-relapsing progressive MS compared to RRMS [32,51]. Furthermore, the present study confirmed the known strong intraindividual correlation between NfL concentrations in CSF and serum, highlighting the potential as a serum biomarker for MS [52,53]. Interestingly, concentrations of NfL correlated with those of the glial activation marker GFAP in serum but not in the CSF. Reasons for this divergence are not yet known and require further research. In contrast to neuroaxonal damage, glial activation (as indicated by GFAP and CHI3L1 [54,55,56]) apparently does not differ between MRZR+ and MRZR- PPMS patients. Of the CSF biomarkers studied in PPMS, only GFAP in serum seems to be clearly age-related. This observation has been made before and needs consideration in future studies analyzing GFAP in serum [24].

### 3.4. Limitations

Important limitations of this study are the retrospective design, some incomplete data subsets (especially regarding follow-up data), lack of data on B cell numbers and their subsets in the CSF, as well as radiological data regarding disease activity [1]. Other B cell cytokines of potential interest were not studied, e.g., APRIL [37,40]. Another study limitation is the lack of serology data. Local infection and vaccination rates can influence the MRZR results, as has exemplarily been shown for the rubella virus in Cuba [57].

### 3.5. Conclusions Summary

In PPMS patients, MRZR positivity might be associated with more pronounced neuroaxonal damage, represented by higher NfL levels in the CSF. However, MRZR status seems neither to indicate clinical disease severity nor disease progression. No support was found for a link between MRZR status and either B cell biomarkers or glial activation markers.

## Figures and Tables

**Table 1:** Clinical features.

	MRZR+ PPMS *(n = 37)*	MRZR- PPMS *(n = 44)*	Comparison Statistics
Sex, females in %	56.3	52.8	n.s. *(data available for n = 81)*
Mean age in years at the time of symptom onset (range; SD)	43.7 (24–62; 10.3)	42.8 (15–60; 10.2)	n.s. *(n = 77; missing data: n = 4)*
Age in years at the time of LP	51.6 (32–78; 11.8)	50.0 (25–70; 10.1)	n.s. *(n = 81)*
Disease duration in years at the time of LP	6.6 (1–39; 7.9)	8.0 (1–27; 7.5)	n.s. *(n = 77; missing data: n = 4)*
EDSS at the time of LP	4.8 (2.0–8.5; 1.6)	4.7 (1.5–8.0; 1.8)	n.s. *(n = 73; missing data: n = 8)*
EDSS at last follow-up	5.6 (4.0–7.0; 1.2)	5.7 (1.5–8.0; 2.2)	n.s. *(n = 20; missing data: n = 61)*
Individual EDSS progression between LP and last follow-up	1.0 (0–2.5; 1.0)	1.0 (−0.5–4.5; 1.6)	n.s. *(n = 20; missing data: n = 61)*
Frequency of a clinical progressive disease within the last two years prior to LP in %	87.5	88.6	n.s. *(n = 67; missing data: n = 14)*

MRZR: measles, rubella, and varicella zoster virus reaction; MRZR+: at least two positive MRZ antibody indices; MRZR-: no more than one positive MRZ antibody index; PPMS: primary progressive multiple sclerosis; SD: standard deviation; LP: lumbar puncture; EDSS: Expanded Disability Status Scale; n.s.: not statistically significantly different. Frequencies of available data did not differ significantly between MRZR+ and MRZR- patients in all parameters.

**Table 2:** Cerebrospinal fluid (CSF) routine parameters.

	MRZR+ PPMS *(n = 37)*	MRZR- PPMS *(n = 44)*	Comparison Statistics
Mean leucocyte count in cells/µL (range; SD)	3.0 (0–11; 2.4)	5.7 (0–43; 8.5)	n.s. *(data available for n = 68; missing data: n = 13)*
Total protein in mg/L	522.0 (278.0–1200.0; 220.5)	520.2 (151.0–1410.0; 220.2)	n.s. *(n = 68; missing data: n = 13)*
Q_Alb_ × 10^−3^	6.8 (2.9–17.8; 3.2)	6.2 (2.7–20.1; 3.0)	n.s. *(n = 68; missing data: n = 13)*
Frequency of an elevated Q_Alb_ in %	38.1	31.8	n.s. *(n = 68; missing data: n = 13)*
Intrathecal synthesis of IgG (IgG_loc_)	14.2 (0–50.6; 18.0)	24.5 (0–149.8; 37.5)	n.s. *(n = 32; missing data: n = 49)*
Frequency of an intrathecal IgG synthesis in %	42.9	40.9	n.s. *(n = 43; missing data: n = 38)*
Frequency of an intrathecal IgA synthesis in %	9.5	0	n.s. *(n = 43; missing data: n = 38)*
Frequency of an intrathecal IgM synthesis in %	14.3	22.7	n.s. *(n = 43; missing data: n = 38)*
IgG index	0.88 (0.5–1.40; 0.32)	1.03 (0.40–2.60; 0.52)	n.s. *(n = 32; missing data: n = 49)*
Frequency of positive OCB in %	89.2	95.5	n.s. *(n = 81)*
Lactate in mmol/L	1.9 (1.4–2.9; 0.5)	1.6 (1.2–2.3; 0.3)	n.s. *(n = 25; missing data: n = 56)*

CSF: cerebrospinal fluid; MRZR: MRZ reaction; MRZR+: at least two positive MRZ antibody indices; MRZR-: not more than one positive MRZ antibody index; PPMS: primary progressive multiple sclerosis; SD: standard deviation; n.s.: statistically significantly different; Q_Alb:_ albumin quotient; IgG/A/M: immunoglobulin G/A/M; OCB: oligoclonal bands. Frequencies of available data did not differ significantly between MRZR+ and MRZR- patients in all parameters.

**Table 3:** B cell biomarkers in CSF.

	MRZR+ PPMS *(n = 37)*	MRZR- PPMS *(n = 44)*	Comparison Statistics
Mean CSF concentration of **BAFF** in pg/mL (range; SD)	92.7 (24.7–387.5; 68.8)	103.6 (18.5–335.5; 77.7)	n.s. *(data available for n = 75; missing data: n = 6)*
**CXCL-13** in pg/mL	18.6 (0–111.0; 26.8)	33.4 (0–295.0; 58.3)	n.s. *(data available for n = 76; missing data: n = 5)*
**sBCMA** in pg/mL	587.7 (149.9–1931.5; 414–8)	658.8 (0–2051.5; 524.2)	n.s. *(data available for n = 59; missing data: n = 22)*
**sTACI** in pg/mL	155.7 (0–1662.5; 311.5)	175.3 (0–1686.6; 339.9)	n.s. *(data available for n = 57; missing data: n = 24)*

CSF: cerebrospinal fluid; MRZR: MRZ reaction; MRZR+: at least two positive MRZ antibody indices; MRZR-: no more than one positive MRZ antibody index; PPMS: primary progressive multiple sclerosis; CSF: cerebrospinal fluid; BAFF: B cell-activating factor; SD: standard deviation; n.s.: not statistically significantly different; CXCL-13: chemokine CXC ligand 13; sBCMA: soluble B cell maturation antigen; sTACI: soluble transmembrane activator and CAML interactor. Detection rates of all parameters did not differ significantly between MRZR+ and MRZR- patients.

**Table 4:** Biomarkers of glial activation and neuroaxonal damage.

	MRZR+ PPMS *(n = 37)*	MRZR- PPMS *(n = 44)*	Comparison Statistics
Mean concentration of **CHI3L1** in CSF in ng/mL (range; SD)	236.4 (87.8–453.2; 108.0)	219.8 (71.8–525.0; 112.6)	n.s. *(data available for n = 78; missing data: n = 3)*
**GFAP** in CSF in pg/mL	9807 (1572–21,760; 5420)	9053 (2480–24,120; 4087)	n.s. *(n = 79; missing data: n = 2)*
**GFAP** in serum in pg/mL	142.4 (67.0–391.0; 76.7)	149.0 (35.6–289.0; 62.5)	n.s. *(n = 68; missing data: n = 13)*
**NfL** in CSF in pg/mL	1842 (598–5384; 1124)	1696 (322–12,720; 2090)	*p* = 0.04 *(n = 78; missing data: n = 3)*
**NfL** in serum in pg/mL	34.8 (8.4–291.5; 55.3)	26.1 (5.7–147.6; 26.7)	n.s. *(n = 68; missing data: n = 3)*

MRZR: MRZ reaction; MRZR+: at least two positive MRZ antibody indices; MRZR-: no more than one positive MRZ antibody index; PPMS: primary progressive multiple sclerosis; CHI3L1: chitinase 3-like 1 protein; CSF: cerebrospinal fluid; SD: standard deviation; n.s.: not statistically significantly different; GFAP: glial fibrillary acidic protein, NfL: neurofilament light chain.

## Data Availability

The datasets generated for this study are available on reasonable request to the first author.

## Abbreviations

AI	Antibody index
AIs	Antibody indices
APRIL	A proliferation-inducing ligand
BAFF	B cell-activating factor
BCMA	B cell maturation antigen
CHI3L1	Chitinase-3-like protein 1
CSF	Cerebrospinal fluid
CXCL-13	Chemokine CXC ligand 13
EDSS	Expanded Disability Status Scale
ELISA	Enzyme-linked immunosorbent assay
GFAP	Glial fibrillary acidic protein
IgG/A/M	Immunoglobulin G/A/M
IgG_loc_	Intrathecal synthesis of IgG
LP	Lumbar puncture
MRZ	Measles, rubella, and varicella zoster virus
MRZR	MRZ reaction
MRZR+	Patients with a positive MRZR result defined as at least two positive MRZ antibody indices
MRZR-	Patients with a negative MRZR result defined as no more than one positive MRZ antibody index
MS	Multiple sclerosis
NfL	Neurofilament light chain
*n*.s.	Not statistically significantly different
OCB	Oligoclonal IgG bands
OCB+	Patients with at least two positive oligoclonal IgG bands in the CSF
OCB-	Patients with not more than one oligoclonal IgG band in the CSF
PPMS	Primary progressive multiple sclerosis
Q_Alb_	Albumin quotient
RRMS	Relapsing–remitting multiple sclerosis
sBCMA	Soluble B cell maturation antigen
SD	Standard deviation
SIMOA	Single molecule array
sTACI	Soluble transmembrane activator and CAML interactor
TACI	Transmembrane activator and CAML interactor
